# Gene expression profiling of whole blood: Comparison of target preparation methods for accurate and reproducible microarray analysis

**DOI:** 10.1186/1471-2164-10-2

**Published:** 2009-01-05

**Authors:** Kristina Vartanian, Rachel Slottke, Timothy Johnstone, Amanda Casale, Stephen R Planck, Dongseok Choi, Justine R Smith, James T Rosenbaum, Christina A Harrington

**Affiliations:** 1 Gene Microarray Shared Resource, Oregon Health & Science University, Portland, OR, USA; 2 Casey Eye Institute, Oregon Health & Science University, Portland, OR, USA; 3 Department of Cell & Developmental Biology, Oregon Health & Science University, Portland, OR, USA; 4 Department of Medicine, Oregon Health & Science University, Portland, OR, USA; 5 Department of Public Health & Preventive Medicine, Oregon Health & Science University, Portland, OR, USA; 6 Vaccine and Gene Therapy Institute, Oregon Health & Science University, Portland, OR, USA

## Abstract

**Background:**

Peripheral blood is an accessible and informative source of transcriptomal information for many human disease and pharmacogenomic studies. While there can be significant advantages to analyzing RNA isolated from whole blood, particularly in clinical studies, the preparation of samples for microarray analysis is complicated by the need to minimize artifacts associated with highly abundant globin RNA transcripts. The impact of globin RNA transcripts on expression profiling data can potentially be reduced by using RNA preparation and labeling methods that remove or block globin RNA during the microarray assay. We compared four different methods for preparing microarray hybridization targets from human whole blood collected in PAXGene tubes. Three of the methods utilized the Affymetrix one-cycle cDNA synthesis/in vitro transcription protocol but varied treatment of input RNA as follows: i. no treatment; ii. treatment with GLOBINclear; or iii. treatment with globin PNA oligos. In the fourth method cDNA targets were prepared with the Ovation amplification and labeling system.

**Results:**

We find that microarray targets generated with labeling methods that reduce globin mRNA levels or minimize the impact of globin transcripts during hybridization detect more transcripts in the microarray assay compared with the standard Affymetrix method. Comparison of microarray results with quantitative PCR analysis of a panel of genes from the NF-kappa B pathway shows good correlation of transcript measurements produced with all four target preparation methods, although method-specific differences in overall correlation were observed. The impact of freezing blood collected in PAXGene tubes on data reproducibility was also examined. Expression profiles show little or no difference when RNA is extracted from either fresh or frozen blood samples.

**Conclusion:**

RNA preparation and labeling methods designed to reduce the impact of globin mRNA transcripts can significantly improve the sensitivity of the DNA microarray expression profiling assay for whole blood samples. While blockage of globin transcripts during first strand cDNA synthesis with globin PNAs resulted in the best overall performance in this study, we conclude that selection of a protocol for expression profiling studies in blood should depend on several factors, including implementation requirements of the method and study design. RNA isolated from either freshly collected or frozen blood samples stored in PAXGene tubes can be used without altering gene expression profiles.

## Background

Gene expression profiling of RNA extracted from peripheral blood is an informative method used to identify biomarkers, examine disease states, and investigate immune responses. However, the relatively high proportion of globin messenger RNA present in total RNA extracted from whole blood can reduce the efficacy of the microarray assay by interfering with the detection of less abundant gene transcripts [1-3]. Furthermore, it has been observed that, when compared with a leukocyte isolation protocol, RNA isolated directly from whole blood is associated with increased noise and reduced sensitivity in the gene expression assay [2,4,5] (K. Vartanian, C. Harrington, unpublished observations).

Common laboratory practice often includes fractionation of whole blood components prior to RNA extraction. The process allows for the removal of the red blood cells from whole blood isolations and facilitates the study of more homogeneous cell populations. Depending on the fractionation method selected, partial or complete removal of reticulocytes, the primary source of globin RNA, may be achieved. Despite the obstacle posed by globin RNA contamination, however, there are compelling reasons to study gene expression from whole blood rather than from subpopulations such as neutrophils. First, to capture expression profiles that accurately reflect the transcriptome at time of blood collection and to minimize sample handling artifacts, it is preferable to avoid additional processing steps as some degree of cell activation is inevitable during cell fractionation. Second, even when fractionation steps are performed, α and β globin mRNA transcripts are often the most abundant transcripts present in total RNA extracted from leukocyte-enriched populations [2] and the fractionation itself can contribute to increases in sample-to-sample variability in the microarray assay [5]. Third, there are a myriad of populations within whole blood: neutrophils, T cells, B cells, NK cells, monocytes, eosinophils, basophils, dendritic cells, and subsets for each of the above. While some studies may require analysis of individual cell types, if all these populations can be studied together, the cost savings is substantial. Finally, working with whole blood saves time and, if specimens are being collected at multiple study sites, the methodology facilitates a uniformity that is diminished with each additional step in the processing of the blood.

Several commercially available methods for reducing the impact of globin RNA transcripts in the microarray expression assay have been developed. One approach involves the removal of α and β globin RNAs from total RNA by selective hybridization and magnetic bead separation [6] prior to amplification and labeling of microarray targets. The Ambion GLOBINclear kit utilizes this pre-labeling method of globin transcript depletion. Using a different approach, Affymetrix and PreAnalytiX have developed a protocol in which α- and β-globin mRNA mRNAs are selectively blocked during the cDNA synthesis step of the microarray target preparation protocol with a mixture of peptide nucleic acids (PNAs) complementary to globin transcripts. Recently, a third method has been released by NuGEN Technologies that does not directly reduce globin RNA transcripts but rather uses a proprietary technology to produce a single-stranded cDNA microarray target [7]. The cDNA targets might be less prone to the cross-hybridization artifacts seen with cRNA targets [8], thus reducing the impact of highly abundant transcripts present in the target and increasing assay sensitivity [9].

Each method has been demonstrated to produce array data in which an increased number of non-globin gene transcripts are detected [1,10], and Debey et al[11] have shown that another globin RNA reduction method also improves microarray performance with whole blood samples. Currently, however, there is limited information available directly comparing these methods or examining the accuracy of microarray profiles generated using the individual methods. In this study we examine the sensitivity, reproducibility and concordance of microarray data produced by targets generated from globin RNA-depleted and non-depleted total RNA using two different target labeling methods: the Affymetrix one-cycle target labeling (standard laboratory method) protocol and the NuGEN Ovation protocol. Quantitative RT-PCR of total blood RNA was used to generate an independent measure of mRNA levels from genes of the human NF-κB signaling pathway in order to evaluate the accuracy of the microarray results. The results of this study allow us to identify optimal methods for preparing microarray targets from blood RNA for expression analysis. In addition we compare expression profiles of fresh and frozen whole blood samples collected in PAXGene tubes to assess the impact freezing may have on expression profiling.

## Results and discussion

RNA isolated directly from whole blood and processed using standard target preparation methods produces gene expression profiles with increased noise and reduced sensitivity compared with total RNA isolated after various leukocyte isolation protocols. However, the advantages of profiling gene expression from whole blood are compelling: 1) cell fractionation steps that may artifactually alter gene expression patterns are avoided; 2) easy and rapid isolation of RNA from whole blood facilitates clinical studies; 3) even rare subpopulations of cells such as dendritic cells or eosinophils remain included in the transcriptome; and 4) costs are vastly reduced. To increase assay sensitivity and reproducibility when profiling RNA extracted from whole blood, it is necessary to employ a method to reduce the impact of highly abundant globin mRNAs on target hybridization with the microarray. In this study, four methods for RNA preparation and labeling were examined to determine which protocol produced the most sensitive and reproducible results when blood RNA isolated from PAXGene tubes was used as input. In addition, since the option to freeze a whole blood sample allows samples to be collected over time and at different study sites prior to processing, the effects of freezing blood samples were examined by comparing data generated from both fresh and frozen specimens. Freezing blood samples after collection has the advantage of allowing batching of samples for RNA extraction and simplifying the blood collection protocol for multicenter studies.

### Methods summary

**Figure 1:**
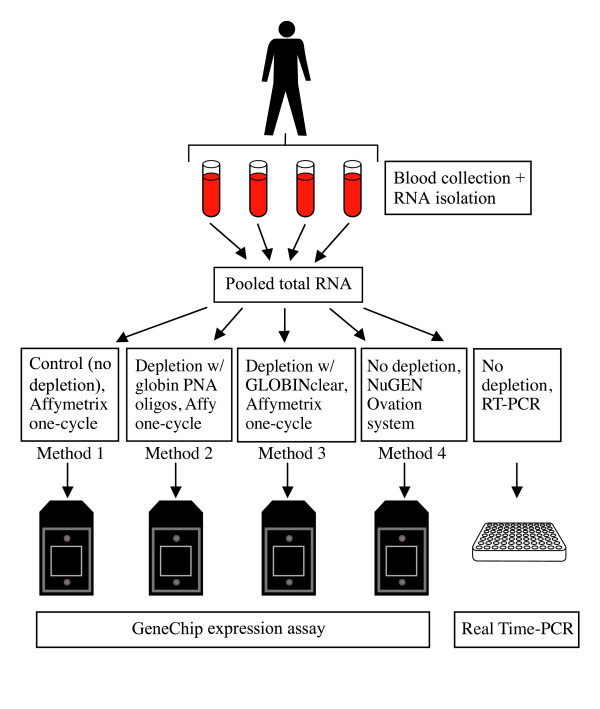
**Flow diagram of whole blood methods comparison study**. A total of 8 PAXGene blood tubes were collected from each donor: 4 tubes were processed on day of collection and 4 were frozen for later processing. RNA was isolated and pooled prior to microarray target labeling with one of four different methods. Microarray results were compared among methods and with quantitative RT-PCR for each donor sample.

Blood samples were collected from healthy, human donors in PAXGene tubes; RNA was isolated either on day of collection or after freezing and storage. Four different methods of microarray target preparation for whole blood RNA samples were examined (Figure 1). Three of the methods used total RNA extracted from whole blood as the starting sample for mRNA amplification and target labeling: Affymetrix one-cycle target labeling (Method 1: no depletion_Affymetrix); Affymetrix one-cycle target labeling with globin PNAs added during cDNA synthesis (Method 2: Globin PNAs_Affymetrix); and NuGEN Ovation system v1 (Method 4: no depletion_NuGEN). In the other method tested, total RNA was treated with Ambion GLOBINclear to reduce globin transcripts prior to labeling with the Affymetrix one-cycle labeling protocol (Method 3: GLOBINclear_Affymetrix). The Affymetrix one-cycle target labeling protocol produces biotin-labeled, amplified cRNA; the NuGEN protocol produces biotin-labeled, amplified cDNA targets.

### Input RNA and target quality assessment

**Figure 2:**
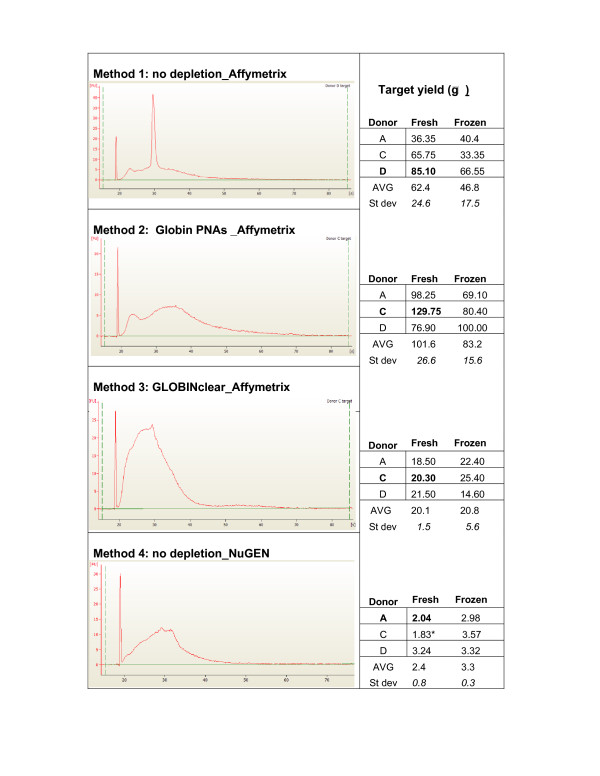
**Quality assessment of cRNA and cDNA targets**. A representative electropherogram (Bioanalyzer trace) of targets produced with each labeling method is shown. For each method, target yields for all samples are listed to the right of the trace. Values in bold text correspond to the sample profiled in the adjacent electropherogram trace.

The quality of total RNA prepared from fresh and frozen PAXGene tubes and from GLOBINclear-treated RNA was assessed using standard UV spectrophotometry and by examining electropherogram images generated with the Agilent Bioanalyzer. Input RNA was of high quality for both fresh and frozen samples (average RIN score 8.9 and 8.8, respectively) with a slight reduction in average quality metrics for GLOBINclear-treated samples (average RIN score 8.6 and 8.5). Reductions in RIN scores after GLOBINclear treatment of whole blood RNA were also observed by Liu et al. [1]. Each amplified target was assessed for cRNA or cDNA quality by examining electropherogram images and for yield using UV spectrophotometry. Results are summarized in Figure 2. For targets generated with Method 1 (no depletion_Affymetrix) the traces display a sharp peak characteristic of highly abundant globin cRNA. This peak is reduced in cRNA targets produced with Methods 2 (Globin PNAs_Affymetrix) and 3 (GLOBINclear_Affymetrix). The absence of the globin RNA-associated peak in targets produced following GLOBINclear treatment or with globin RNA blockage by PNA oligos during cDNA synthesis is consistent with the results reported by Liu et al[1] in their study evaluating these methods. The Bioanalyzer trace of targets produced with Method 3 (GLOBINclear_Affymetrix) displays a pattern characteristic of mild RNA degradation (a left-shifted trace); this may be a result of the extra handling steps required by the GLOBINclear treatment. The cDNA trace for Method 4 (no depletion_NuGEN) targets does not show a discrete peak, suggesting that globin RNA amplification may also be reduced with this method.

Using recommended RNA inputs for each method, average cRNA target yield ranged from 20 μg to 102 μg. The average yield with Method 1 (no depletion_Affymetrix) using RNA from either fresh or frozen PAXGene tubes is typical of yields obtained in our laboratory with this method. We speculate that the high yields for Method 2 (Globin PNAs_Affymetrix) relative to the other Affymetrix one-cycle target labeling methods are, at least partially, the result of the higher input RNA amount (5 μg) required by the Method 2 protocol. The GLOBINclear-treated RNA produced the lowest cRNA yields with the Affymetrix one-cycle labeling protocol, as has been observed previously with this method [1]. The NuGEN method generated an average cDNA target yield of 2.8 μg from an RNA input of 30 ng, which is within the reported yield range for this assay. Target yields for all methods tested in this study were sufficient for performing microarray analysis.

### Array hybridization performance

Targets produced with each labeling method were hybridized with the GeneChip Human Focus array. Target amounts and array processing steps were performed according to the manufacturers' recommendations for the labeling method and the array format. Overall performance in the array assay was examined in three ways: 1) individual array performance metrics (generated with MAS 5.0); 2) data visualization for assessment of data distribution and variation; and 3) correlation of detected transcripts among targets generated with each method.

Array performance metrics (Table 1) indicate that labeling Method 2 (Globin PNAs_Affymetrix) produced targets with the highest transcript detection efficiency (% present) among the methods tested. The next most sensitive result was seen with Method 3 (GLOBINclear_Affymetrix). Background levels were similar among the targets produced with the three methods utilizing the Affymetrix one-cycle cDNA/IVT labeling protocol. The laboratory standard method of target preparation (no depletion_Affymetrix) demonstrates that lowest transcript detection when used with whole blood RNA. The NuGEN Ovation labeling method produced lower overall signal and lower background on the arrays. Actin 3'/5' ratios measured with this method are 5–9 fold higher than with the other labeling methods. This pattern is consistent with previous reports describing the Ovation system [7,12] and, taken together with the low GAPDH ratios produced with this Method, is not likely to represent poor quality RNA or targets. Only one microarray assay was flagged for poor performance in this study: Sample A, Method 1 (no depletion_Affymetrix). (In our estimation, the performance characteristics of Sample A with Method 1 indicate a technical outlier whose underperformance is not a result of sample source or labeling method type.)

**Table 1:** GeneChip Array performance metrics

**Sample**	**Bg**	**% P**	**Mean Signal**	**Actin Intensity**	**Actin 3'/5' Ratio**	**GAPDH Intensity**	**GAPDH 3'/5' Ratio**
**METHOD 1**							

A	54.3	**48.3%**	95.5	751.4	**7.6**	410.4	**5.9**
C	50.3	53.1%	123.3	3437.5	1.4	988.1	1.8
D	47.8	52.2%	107.5	3692.0	1.1	1216.7	1.1
A frozen	49.0	51.2%	95.0	2641.6	1.4	1073.3	1.2
C frozen	48.7	54.0%	117.7	3618.0	1.2	1324.7	1.2
D frozen	49.7	51.3%	98.6	2933.7	1.1	1005.6	1.5
Average*	*50.1*	*52.4%*	*106.3*	*2845.7*	*1.2*	*1003.1*	*1.4*

**METHOD 2**							

A	58.2	58.9%	270.4	8145.6	1.5	3492.0	0.9
C	52.7	58.7%	220.0	6812.9	1.5	2742.4	1.0
D	62.2	55.9%	179.7	6953.0	1.1	2697.9	1.0
A frozen	53.1	57.2%	177.2	5616.2	1.5	2336.8	1.1
C frozen	56.1	58.5%	195.4	6458.0	1.2	2580.7	0.9
D frozen	55.2	58.3%	196.8	6952.1	1.2	3016.1	1.0
*Average*	*56.2*	*57.9%*	*206.6*	*6823.0*	*1.3*	*2811.0*	*1.0*

**METHOD 3**							

A	53.2	56.9%	155.8	2014.2	3.2	680.1	8.3
C	51.3	56.1%	145.1	4514.5	1.1	689.9	4.3
D	51.7	55.7%	144.5	3492.7	1.7	676.4	8.0
A frozen	46.5	56.6%	136.7	3400.5	2.0	1442.0	1.4
C frozen	49.5	55.1%	129.7	1491.0	3.9	583.8	8.9
D frozen	45.6	56.3%	132.2	3598.3	1.2	678.7	2.9
*Average*	*49.6*	*56.1%*	*140.7*	*3085.2*	*2.2*	*791.8*	*5.6*

**METHOD 4**							

A	41.8	55.8%	97.0	2057.3	7.2	301.5	1.5
C	30.9	53.4%	63.6	1658.4	10.7	161.3	1.6
D	31.2	55.7%	82.4	2039.6	10.2	259.4	1.9
A frozen	30.8	53.8%	65.2	1577.0	15.5	183.4	2.9
C frozen	34.8	53.3%	73.2	1753.5	11.1	190.2	2.1
D frozen	37.9	53.3%	79.3	1838.4	10.6	256.3	1.8
*Average*	*34.6*	*54.2%*	*76.8*	*1820.7*	*10.9*	*225.4*	*2.0*

Microarray targets prepared from donors A, C, and D were hybridized to the GeneChip Human Focus Array. Overall performance values determined with unscaled MAS 5.0 analysis of each array are listed. Bold font indicates metric values that fall below empirical quality control standards established in our laboratory. All arrays passed post-hybridization visual inspection. *Average values for Method 1 do not include sample A which was flagged as a low performance outlier.

**Figure 3:**
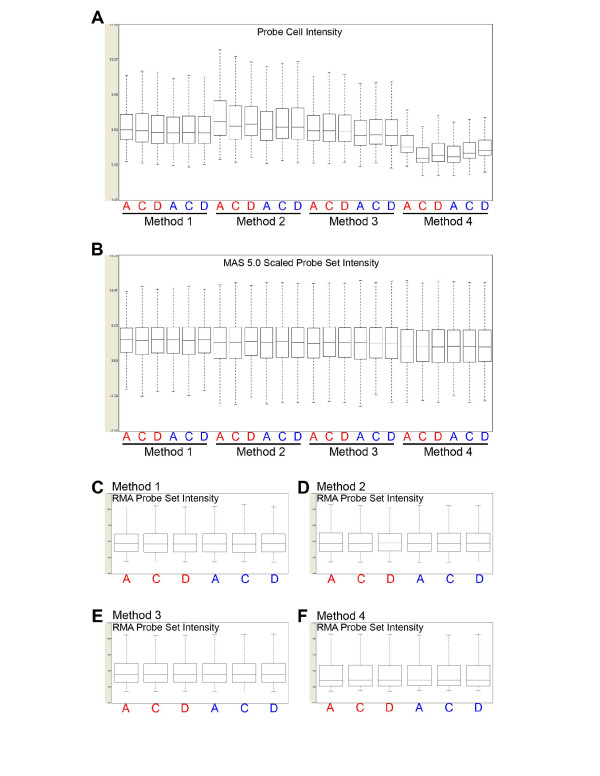
**Box plots of raw probe cell and normalized probe set signal intensities**. All box plots were generated with Affymetrix Expression Console. A. Probe cell intensity box plot generated from CEL file probe cell intensity values for all targets; B. Box plot of globally scaled, MAS 5.0 summarized probe set signals for all targets. C. Box plot of RMA summarized probe set signals for Method 1 targets; D. Box plot of RMA summarized probe set signals for Method 2 targets; E. Box plot of RMA summarized probe set signals for Method 3 targets; F. Box plot of RMA summarized probe set signals for Method 4 targets. Donor source is indicated on the X-axis: red text indicates RNA isolation from PAXGene tubes on the day of collection, blue text indicates RNA isolation from PAXGene tube following freezing.

Examination of probe cell intensity distributions prior to normalization shows that overall signal distribution patterns (Figure 3A) varied with the labeling method. Within-method variation is low across methods, with the exception of Method 4 (no depletion_NuGEN) which showed somewhat greater variation than the other methods. Probe cell intensity range, however, is notably different among methods, with Method 2 (Globin PNAs_Affymetrix) showing the greatest range and Method 4 (no depletion_NuGEN) the lowest. Because of the large differences in detection sensitivity and gene expression patterns among the four labeling methods, likely due to differences in globin transcript levels and target nucleic acid types (i.e., cRNA vs cDNA), we applied model-based normalization algorithms to within-method array data as well as across the entire data set. Following gene summarization and MAS scaling (Figure 3B) or RMA quantile normalization (Figure 3C–F), within-method variation is not pronounced for any of the labeling methods, although, Method 1(no depletion_Affymetrix) showed a compressed probe set signal intensity range compared to the other labeling methods. Interestingly, in this study, the sample-to-sample variation observed with Method 1 (no depletion_Affymetrix) is reduced compared with other microarray studies in our laboratory using this method with RNA extracted from whole blood or Ficoll-Hypaque-isolated PBMCs. Possible explanations for the reduced variation in the present study include 1) use of highly standardized blood collection and RNA processing protocols, with all target labeling performed in a single batch, 2) low number of independent samples in this study is insufficient to detect typical variation, 3) blood samples were only collected from healthy donors. This latter possibility is consistent with the report of Whitney et al [13] in which a reduced variation in gene expression patterns in blood from healthy individuals compared to patients with bacterial infection was observed.

Sample-to-sample variation was also examined by measuring similarity among expression profiles from different donor samples processed with the same target preparation method. A high within-method correlation (average r = 0.98) was measured for Methods 1 (no depletion_Affymetrix; excluding the Sample A outlier), 2 (Globin PNAs_Affymetrix) and 4 (no depletion_NuGEN). Method 3 (GLOBINclear_Affymetrix) again showed somewhat poorer performance (average r = 0.96).

### Comparison of globin transcript detection

**Figure 4:**
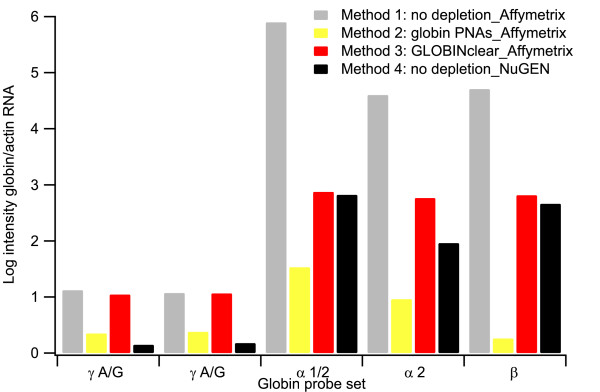
**Relative signal levels for globin transcripts in microarray data**. The average log_2 _intensity ratio of globin RNA probe sets to the control actin probe set is shown. The Human Genome Focus Array contains two probe sets that hybridize with γ hemoglobin RNA, two probe sets that hybridize with α-globin RNA, and one probe set that hybridizes with β-globin RNA.

We assessed the extent of globin transcript depletion in the microarray assay with each labeling method by examining α- and β-globin mRNA signal intensities normalized to actin mRNA signal. All of the globin mRNA depletion methods resulted in decreased α- and β-globin mRNA transcript measurements relative to Method 1 (no depletion_Affymetrix) targets. Method 2 (Globin PNAs_Affymetrix) generated targets that showed the greatest reduction in globin mRNAs (Figure 4). Interestingly, Methods 2 (Globin PNAs_Affymetrix) and 4 (no depletion_NuGEN) also resulted in reduced hybridization signal with γ globin mRNA probe sets on the Affymetrix arrays, but Method 3 (GLOBINclear_Affymetrix) showed no reduction in these signals relative to the standard Method 1 (no depletion_Affymetrix) results.

### Quantitative RT-PCR validation

Expression levels of a panel of 84 genes from the NF-κB pathway plus 5 housekeeping genes were measured by quantitative RT-PCR using total RNA from two of the individuals profiled in the microarray component of this study. The NF-κB pathway was chosen because of biological interest to study authors and high overlap of panel genes with genes represented on the Human Focus array. RNA prepared from both frozen and unfrozen PaxGene tubes was analyzed for each donor. All of the genes measured in the RT-PCR assay were scored as present in the microarray profiles produced with targets from one or more of the methods examined in this study.

**Figure 5:**
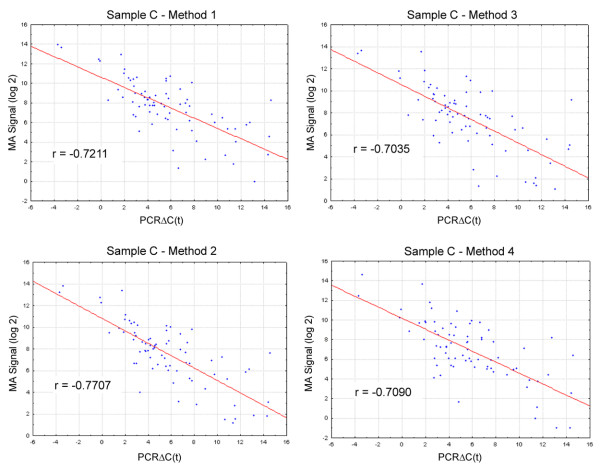
**Comparison of microarray and quantitative RT-PCR results for genes of NF-κB pathway**. Scatter plots of MAS5.0 scaled probe set intensities measured on the GeneChip Human Genome Focus Array and real-time PCR ΔCt values for 75 genes of the NF-κB pathway. **A. **Sample C, microarray targets prepared with Method 1; **B. **Sample C, microarray targets prepared with Method 2; **C. **Sample C, microarray targets prepared with Method 3; **D. **Sample C, microarray targets prepared with Method 4. Microarray data was analyzed and scaled with MAS 5.0 and RT-PCR data was normalized by subtracting the mean Ct value for five housekeeping genes (β_2_-microglobin, hypoxanthine phosphoribosyltransferase 1, ribosomal protein L13a, glyceraldehyde-3-phosphate dehydrogenase, and β-actin) to yield ΔCt values. Pearson correlation coefficients are reported for each comparison.

Comparison of the RT-PCR data with array profiles for 75 matched genes demonstrated that Method 2 (Globin PNAs_Affymetrix) produced expression data that most closely correlates with the RT-PCR results, with an average Pearson correlation of 0.77 (Figure 5 and Table 2). Surprisingly, despite the presence of abundant levels of globin transcripts, the targets produced by the one-cycle cDNA synthesis protocol with Affymetrix IVT (no depletion_Affymetrix), show the next highest correlation with the RT-PCR results. In their comparison of differential gene expression results, Barker et al [8] reported that cDNA targets prepared with the Ovation v1 method more closely tracked with quantitative RT-PCR measurements than cRNA targets prepared with the traditional one-cycle cDNA/IVT method (Method 1 in the present study). It is difficult to interpret the significance of this observation in the context of our results as RNA source, study design, PCR primer selection rationale, and analysis methodology differ significantly between the two studies. However, taken together, the results may indicate that absolute measurements of gene expression and relative measurements of differential gene expression may not be co-ordinately affected by a particular target preparation method.

**Table 2:** Correlation (r) of quantitative RT-PCR and microarray data for each Method

**Donor**	**Method 1** **No depletion_Affy**	**Method 2** **Globin PNAs_Affy**	**Method 3** **GLOBINclear_Affy**	**Method 4** **No depletion_NuGEN**
C	-0.72	-0.77	-0.70	-0.71
D	-0.75	-0.76	-0.69	-0.70
C frozen	-0.77	-0.77	-0.73	-0.71
D frozen	-0.75	-0.78	-0.69	-0.70

Correlation of RT-PCR and microarray expression measurements for 75 genes of the NF-κB pathway is shown: ΔC(t) values for RT-PCR and scaled, MAS 5.0 gene level signals for microarray data. The Pearson product moment correlation coefficient is reported for each target-labeling method examined in this study. RNA samples from donors C and D were prepared from either fresh or frozen PAXGene tubes.

### Cross-method comparison of expression profiles

**Figure 6:**
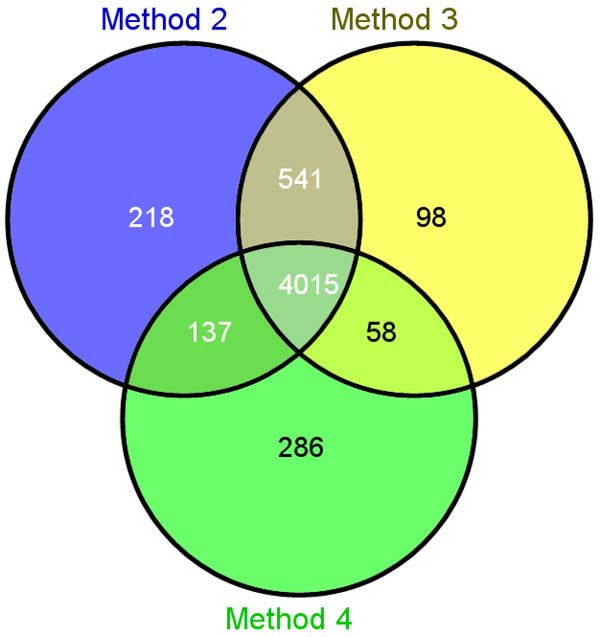
**Venn diagram of transcript detection among globin RNA reduction methods**. Gene expression lists for each target preparation method were determined by filtering for all probe sets scored as Present (p-value < 0.05) in 4 out of 6 microarray assays (donors A, C and D). Overlap of detected transcripts is shown for Method 2 (Globin PNAs_Affymetrix), Method 3 (GLOBINclear_Affymetrix) and Method 4 (no depletion_NuGEN).

Differences among transcriptome profiles generated with each method were examined to assess concordance among methods. Transcriptome patterns were identified for each labeling method by filtering for probe sets with a minimum detection p-value of 0.05 in at least 4 of the 6 within-method array assays using gene level expression data generated with MAS 5.0. Overlap among these transcript lists for Method 2 (Globin PNAs_Affymetrix), Method 3 (GLOBINclear_Affymetrix) and Method 4 (no depletion_NuGEN) is illustrated in the Venn diagram in Figure 6. By far, the majority of transcripts detected with any one method were also detected by each of the other globin-signal reduction methods (4015 probe sets; 46% of total, non-control probe sets on the Human Genome Focus Array). However, over 700 probe sets were only detected with two of the methods and both Methods 2 (Globin PNAs_Affymetrix) and 4 (no depletion_NuGEN) uniquely detected a few hundred additional transcripts. Most transcripts detected with Method 1 (no depletion_Affymetrix) were included in the set of transcripts detected with Method 2 (Globin PNAs_Affymetrix) (98.6%); only 40 probe sets met the detection filtering criteria exclusively in Method 1 (no depletion_Affymetrix) samples, all of which were associated with low probe set signal intensities. It is likely that at least some of the low signal probe sets scored as present with a single target preparation method represent false positives or low abundance transcripts at the borderline for detection. Only Method 4 (no depletion_NuGEN) had a significant portion of uniquely detected transcripts with probe set signal intensities above the lowest quartile of detected transcript intensities.

Overall, the vast majority of gene transcripts detected with each method were also detected by the other three methods. The highest expression concordance was observed between Methods 1 (no depletion_Affymetrix) and 2 (Globin PNAs_Affymetrix). The Globin PNAs_Affymetrix method and the no depletion_NuGEN method each detect a significant number of gene transcripts (~3% of all transcripts represented on the Human Genome Focus array) not detected by any of the other methods tested in this study. Four of the transcripts detected with only one labeling method (3 in no depletion_NuGEN and 1 one in Globin PNAs_Affymetrix) correspond to genes whose transcription was confirmed in the quantitative RT-PCR analysis. This observation suggests that both the no depletion_NuGEN and the Globin PNAs_Affymetrix method produce microarray data that accurately measures gene expression not detected by the other methods.

### Impact of freezing whole blood in PAXGene tubes

Microarry performance of RNA extracted from blood collected in PAXGene tubes immediately following a 2 hr room temperature incubation or after a 2 hr incubation and freezing at -80°C was compared. Correlation between array data from fresh and frozen PAXGene preparations from each donor was high across all samples prepared with Methods 1 (no depletion_Affymetrix), 2 (Globin PNAs_Affymetrix) or 4 (no depletion_NuGEN), with the exception of donor A using Method 1, where one of the microarray assays underperformed (Table 3). Method 3 (Globin PNAs_Affymetrix) resulted in fresh and frozen sample correlations that were somewhat reduced compared to the other target labeling methods. Comparison of the quantitative PCR results of fresh and frozen blood preparations from donors C and D demonstrated a similar high level of correlation (r = 0.98 for donor C and 0.99 for donor B).

**Table 3:** Correlation (r) of array data from donor RNA extracted from fresh versus frozen PAXgene tubes

**Donor**	**Method 1** **No depletion_Affy**	**Method 2** **Globin PNAs_Affy**	**Method 3** **GLOBINclear_Affy**	**Method 4** **No depletion_NuGEN**
A	0.953	0.991	0.959	0.979
C	0.989	0.993	0.959	0.981
D	0.996	0.992	0.988	0.993

Pearson correlation coefficients for each within-method comparison are reported. Microarray data was analyzed and scaled with MAS 5.0.

Our results indicate that freezing blood collected in PAXGene tubes prior to RNA extraction has little impact on the RNA profiles measured in the microarray assay. This observation is consistent with the report of Ovstebo et al[14] in which the expression profile of 11 mRNAs assessed with RT-PCR analysis was not altered by freezing PAXGene tubes prior to RNA extraction.

### Microarray assay reproducibility

Given the overall similarity in fresh and frozen sample pairs as measured in both the microarray and RT-PCR analyses, we elected to use the fresh/frozen pairs for each donor to examine array assay reproducibility among the different assay methods. Method 2 (Globin PNAs_Affymetrix) showed the highest reproducibility in paired donor samples (average r = 0.99). Reproducibility was also high with Methods 1 (no depletion_Affymetrix) and 4 (no depletion_NuGEN) (average r = 0.98), but slightly reduced with Method 3 (GLOBINclear_Affymetrix) (average r = 0.97).

## Conclusion

Removing globin transcripts from cRNA hybridization targets prepared from whole blood samples results in increased sensitivity in microarray expression profiles. Sensitivity is also increased when cDNA hybridization targets prepared from undepleted RNA are used instead of cRNA targets. All of the methods tested in this study generate highly reproducible data, except when total RNA is pre-treated to remove globin transcripts (GLOBINclear_Affymetrix method). Comparison of microarray gene level signals with real-time PCR data suggests that the Globin PNAs_Affymetrix method produces the most accurate microarray results, although, all methods produce data that correlate well with the RT-PCR measurements. Performance results for each method are summarized in Table 4.

**Table 4:** Summary of Methods performance

**METHOD**	**Overall Performance Assessment**
**Method 1 **– Affymetrix one-cycle target labeling of	Lowest sensitivity
whole blood RNA (no depletion_Affymetrix)	Good reproducibility
	Good target integrity

**Method 2 **– Affymetrix one-cycle target labeling of	High sensitivity
whole blood RNA with globin PNAs during cDNA	High reproducibility
synthesis (Globin PNAs_Affymetrix)	Good target integrity

**Method 3 **– Affymetrix one-cycle labeling of whole	High sensitivity
blood RNA treated with Ambion GLOBINclear	Lowest reproducibility
(GLOBINclear_Affymetrix)	Marginal target integrity

**Method 4 **– NuGEN Ovation v1 amplification and	Moderate sensitivity
labeling of whole blood RNA (no depletion_NuGEN)	High reproducibility
	Good target integrity

Before selecting a protocol for use in an expression profiling study of whole blood, however, several factors must be considered.

### Method 1, Affymetrix one-cycle target labeling protocol with no globin RNA depletion produces reliable data but sensitivity is low

#### Advantages

This method required relatively low RNA amounts and did not require any additional processing steps. Overall, correlation to real-time PCR data was second only to the Globin PNAs_Affymetrix method (Method 2).

#### Considerations

It has been well reported that performing microarray profiling of RNA extracted from whole blood using this method without modification results in less sensitive data with high levels of sample-to-sample variability [2,4,5,11]. In the present study the use of this method with whole blood samples resulted in the lowest transcript detection of all the methods tested.

### Method 2, Affymetrix one-cycle target labeling protocol with globin PNAs generates sensitive and reproducible results

#### Advantages

Sensitivity was highest with this method. Additionally, this method also produced the lowest relative α- and β-globin intensities when the signal was normalized to a housekeeping control gene, further confirming the effectiveness of the reduction. Among all protocols tested in this study, the globin PNAs method demonstrated the highest reproducibility when correlation values were measured between donor replicates. Finally, microarray RNA expression values produced with the PNA method showed the highest correlation to RT-PCR measurements of a selected gene panel in the same RNA samples compared to all other methods.

#### Considerations

While targets prepared following the PNA method produced the best overall results in the microarray assay, before selecting this protocol to profile expression patterns of whole blood, it is important to note that, among the protocols examined in this paper, the PNA method is one of the more time-consuming and requires the highest amount of total RNA. Although recent studies in our laboratory show that the PNA method can be successfully applied with RNA inputs as low as 2 μg (unpublished data), the relatively high RNA amounts required by this method could be a confounding factor if the amount of RNA available for expression profiling is low. Furthermore, the PNA sequences supported by Affymetrix for use in this protocol are designed to bind to human α- and β-globin transcripts. Consequently if blood RNA extracted from non-human species is to be analyzed, it would be necessary to design and optimize peptide nucleic acid sequences for the species of interest. Also note that PNA sequences recommended by Affymetrix are no longer sold by Applied Biosystems. The product is now available through Panagene (Yuseong, Daejeon, Korea).

### Method 3, Ambion GLOBINclear processing prior to Affymetrix one-cycle target labeling produces sensitive results but is less reproducible in comparison to the other methods

#### Advantages

The third method examined in this study uses a magnetic-bead based hybridization system to extract globin RNA from whole blood total RNA prior to amplification. Beads can be purchased to bind to species-specific globin transcripts (human and rodent), allowing for increased flexibility. Sensitivity is almost as high as that achieved with the PNA method. Globin probe set intensities, compared to the housekeeping controls, were reduced but not to the level observed when using the PNA method.

#### Considerations

Reproducibility of GLOBINclear targets is lowest of the methods examined. Furthermore, we noted an increase in the ratio of 3' versus 5' housekeeping control probe set intensity, suggesting a slight decrease in the quality of the target prepared using this method. This trend can likely be explained by the increased sample handling required to process the RNA during the bead hybridization and magnetic purification steps. Input requirements are also relatively high for this method, again presenting challenges when sample amounts are limiting. In addition, comparison of microarray expression values generated with this method showed the lowest correlation with the RT-PCR results on the NF-κB pathway genes.

### Method 4, NuGEN Ovation v1 protocol produces somewhat reduced sensitivity measures compared to globin RNA depletion methods but reproducibility is high

#### Advantages

Among the four protocols tested, this labeling protocol requires the lowest amount of total RNA (5–100 ng) and does not have a separate globin RNA reduction step. Instead, the hybridization kinetics of the cDNA target appear to be less affected than cRNA targets by the abundant globin RNA present in whole blood extractions. The α- and β-globin RNA intensity measurements in these targets, compared to a housekeeping control, ranked second to the PNA method indicating a reduction in globin transcript signal compared to the standard labeling method with no globin RNA depletion. In addition, because a separate species-specific globin reduction step is not required, the Ovation method is the most flexible cross-species protocol of the alternative methods tested.

#### Considerations

Microarray expression values generated with this method do not correlate as well with RT-PCR measurements as the Globin PNAs or standard Affymetrix method. In addition, detection sensitivity of targets prepared following the NuGEN Ovation Biotin RNA Amplification and Labeling System v1 protocol is the lowest of the alternative methods tested. However, since we completed this study, NuGEN has released a modified protocol (NuGEN Ovation Whole Blood Labeling System with FLv2) specifically aimed at minimizing the impact of globin transcripts when expression profiling RNA extracted from whole blood. Results from a blood profiling study that used targets generated with the Whole Blood Ovation System show significantly increased transcript detection rates compared to version 1 of this kit (K. Vartanian, R. Slottke, and C. Harrington, unpublished data). It is likely that the Ovation Whole Blood FLv2 system retains the high reproducibility and implementation advantages of the version 1 system while producing microarray data with detection sensitivity similar to the globin PNAs method. In a study of small sample protocols for microarray analysis using non-blood tissues, another aspect of sensitivity, detection of differential expression between samples, was shown to be highest with the Ovation method compared to other small sample protocols and the standard Affymetrix one-cycle labeling method [15].

Our results and observations can be summarized in five main points: (1) protocols designed to minimize the impact of globin transcripts can significantly improve the performance of whole blood-extracted RNA in an expression profiling microarray study; (2) methods to reduce globin RNA prior to target synthesis may require amounts of RNA that are not feasible in some studies; (3) for nonhuman studies, the requirement for species-specific oligonucleotides in globin RNA depletion protocols may limit use; (4) target preparation protocols can be a major source of variability; (5) if precautions are taken to properly stabilize extracted blood, the impact of freezing specimens prior to RNA extraction is minimal.

## Methods

### Blood collection and RNA isolation

Unfractionated whole blood collection and RNA isolation were performed using the PAXgene Blood RNA Isolation System (PreAnalytiX, a Qiagen BD Company, Valencia, CA, USA). PaxGene tubes contain a proprietary reagent to reduce intracellular RNA degradation and minimize induction of gene expression [16]. With the approval of the OHSU Institutional Review Board, blood was collected from four healthy donors using eight PAXGene tubes per individual. After incubation for two hours at room temperature, four PAXGene tubes per donor were transferred to a -80°C freezer. RNA was isolated from the remaining tubes on the day of collection and from the frozen tubes after storage at -80°C for at least one week. DNase-treatment was performed as per PAXGene manufacturer's recommendation. After pooling samples from 4 frozen or 4 fresh PAXGene tubes for each donor, RNA recovery and quality were assessed by examining UV 260/280 absorbance ratios and RNA size distribution on RNA Nano LabChips (Agilent Technologies, Santa Clara, CA, USA) processed on the Agilent 2100 Bioanalyzer. An RNA Integrity Number (RIN) was generated for each Bioanalyzer trace using Expert software (Agilent). For inclusion in this study, a minimum of 12 μg of RNA per donor from both fresh and frozen blood preparations were required in order to have sufficient material for RNA quality assessments and manufacturer recommended inputs for each labeling protocol. Samples from three of the four donors met the quantitative and qualitative criteria.

### Target preparation methods overview

Amplifications and labeling were performed for each donor using one of the following four methods with RNA extracted from fresh and frozen blood samples: Method 1 – Affymetrix one-cycle cDNA synthesis/Affymetrix in vitro-transcription (IVT) on whole blood RNA (no depletion_Affymetrix); Method 2 – Affymetrix one-cycle cDNA/Affymetrix IVT with globin peptide nucleic acids (PNAs) inclusion during cDNA synthesis (Globin PNAs_Affymetrix); and Method 3-RNA pre-treated with Ambion GLOBINclear beads to reduce globin transcripts prior to labeling with the Affymetrix one-cycle cDNA synthesis/Affymetrix IVT (GLOBINclear_ Affymetrix). In the fourth method tested, cDNA target was synthesized from whole blood RNA using the NuGEN Ovation Biotin RNA Amplification and Labeling System v1 (no depletion_NuGEN). Total RNA input amounts were based on manufacturer's recommendations for each method.

### Method 1: Affymetrix one-cycle cDNA synthesis/Affymetrix IVT (no depletion_Affymetrix)

Method 1 employed the standard laboratory method for amplification and labeling of total RNA. Target synthesis was performed following the Affymetrix GeneChip Expression Analysis Technical Manual, rev. 5  with minor modification. Using 1 μg of total RNA as input, messenger RNA was amplified and labeled in two steps. In the first step, mRNA was converted to double-stranded cDNA and purified by phenol-chloroform-isoamyl alcohol extraction and ethanol precipitation. In the second step, amplified and biotinylated cRNA (the target) was produced by in vitro transcription (IVT). Unincorporated nucleotides were removed using the RNeasy Mini kit (Qiagen) followed by an ethanol precipitation of the labeled target.

### Method 2: Affymetrix one-cycle cDNA synthesis/Affymetrix IVT with PNA treatment of total RNA (Globin PNAs_Affymetrix)

The standard Affymetrix amplification and labeling was performed with the following modification: four peptide nucleic acid (PNA) oligonucleotides (Applied Biosystems, Foster City, CA, USA) designed to anneal to human α- and β-globin mRNA were added to the total RNA preparations immediately prior to initiation of the target synthesis step in order to bind globin mRNA and block cDNA synthesis. The PNA oligonucleotide stocks were prepared on day of use at concentrations recommended by Affymetrix . Globin reduction PNA master mix was prepared by adding equal amounts of the globin PNA stocks. Five μg of total RNA were incubated with oligo-dT primer and 1 μl PNA master mix for 10 minutes at 70°C prior to first strand cDNA synthesis; all subsequent steps for target amplification and labeling were as described for Method 1.

### Method 3: Affymetrix one-cycle cDNA synthesis/Affymetrix IVT with GLOBINclear treated total RNA (GLOBINclear_Affymetrix)

Globin mRNA depletion was performed in three steps using the GLOBINclear Kit (Ambion, Austin, TX, USA). First, species-specific biotinylated oligonucleotides complementary to human α- and β-globin mRNA were hybridized with total RNA (5 μg). Second, magnetic streptavidin-coated beads were added to bind the biotinylated-oligonucleotide:globin RNA complexes. Finally, a magnet was used to remove beads with the selectively bound α- and β-globin transcripts. After globin mRNA depletion, RNA quantity was determined by UV absorbance and RNA integrity was assessed using RNA Nano LabChips as described above. RNA recovery following GLOBINclear treatment ranged from 2–5 μg. Target labeling and amplification steps were then performed as described for Method 1.

### Method 4: NuGEN Ovation Biotin RNA Amplification and Labeling System v.1 (no depletion_NuGEN)

Ovation v1 labeling and amplification reagents were obtained from NuGEN Technologies, Inc (San Carlos, CA, USA) and biotinylated cDNA targets were prepared according to manufacturer's instructions. Double-stranded cDNA was synthesized from 30 ng total RNA, followed by a linear isothermal amplification (SPIA Amplification™) to produce single-stranded cDNA (target). A proprietary fragmentation and direct labeling process attached biotin to the amplified target. Target purifications were performed using DNA Clean and Concentrator – 25 (Zymo Research, Orange, CA, USA) and the DyeEx 2.0 Spin Kit (Qiagen).

### Array hybridization and processing

Targets labeled with Affymetrix one-cycle cDNA synthesis/Affymetrix IVT (Methods 1, 2 and 3) were chemically fragmented as per Affymetrix's recommendations and combined with Affymetrix biotinylated hybridization controls (oligomer B2 and cRNAs for BioB, BioC, BioD and CreX) in hybridization buffer. Hybridizations were performed for 16 hours at 45°C after addition of 6.5 μg of target to the GeneChip Human Genome Focus array (Affymetrix), containing 8700 probe sets. Post-hybridization array processing was performed according to manufacturer's recommendations. The distribution of fluorescent material on the processed array was determined using the Affymetrix 3000 GeneArray laser scanner with the 7G upgrade. Image inspection was performed manually immediately following each scan.

For targets labeled with NuGEN Ovation system (Method 4), the hybridization and processing outlined above was performed with the following modification: following NuGEN's recommendations, 1.3 μg of cDNA target were mixed with Affymetrix hybridization controls in hybridization buffer and hybridized with the Human Genome Focus array for 18 hours at 45°C.

### Microarray data quality assessment

The array image scan was processed with Affymetrix Microarray Suite, version 5.0, (MAS 5.0) software. GeneChip expression Arrays contain control probe sets for both spiked and endogenous RNA transcripts (e.g., BioB, BioC, BioD, CreX and species-specific actin and GAPDH). Following absolute analysis of the array pattern with MAS, six values were examined to assess overall assay performance: background, noise, average Signal, % Present, ratio of Signal values for probe sets representing the 5' and 3' ends of actin and GAPDH transcripts, and total Signal for probe sets for BioB, BioC, BioD and CreX. Assays demonstrating poor or marginal performance were flagged.

### Quantitative RT-PCR

Quantitative RT-PCR was performed on RNA from donors C and D. cDNA was prepared from selected RNA samples (1 μg each) with a RT^2 ^PCR Array First Strand Kit (SuperArray Bioscience, Frederick, MD, USA) following the manufacturer's instructions. The product was combined with SuperArray PCR SYBR Green master mix and pipetted into all wells of a 96-well RT^2^Profiler™ PCR Array for the human NF-κB pathway (SuperArray APHS-025A) per the manufacturer's instructions. After 10 min at 95°C to activate the HotStart DNA polymerase, the samples were subjected to 40 cycles of 15 seconds 95°C, 35 seconds at 55°C during which time the SYBR Green fluorescence was recorded, and 30 seconds at 72°C with a Chromo4 thermocycler (BioRad Laboratories, Hercules, CA, USA). Cycle threshold (Ct) values were obtained for each sample and were normalized for each plate by subtracting the mean Ct value for five housekeeping genes (β_2_-microglobin, hypoxanthine phosphoribosyltransferase 1, ribosomal protein L13a, glyceraldehyde-3-phosphate dehydrogenase, and β-actin) to yield ΔCt values.

For comparison of RT-PCR results with the microarray data, 75 genes from the SuperArray NF-κB pathway panel were matched to probe sets on the Affymetrix Human Genome Focus array.

### Data analysis and visualization

Low-level analysis (background correction, normalization, and gene summarization) of microarray data was performed with both Microarray Suite 5.0 (MAS 5.0) [17,18] and Robust Multi-Array Average (RMA) [19,20]. Individual arrays were analyzed and scaled with MAS 5.0 using manufacturer's default thresholds for detection calls. For RMA analysis, arrays were normalized together and in groups based on target labeling method using RMA implemented in Affymetrix Expression Console. All microarray data have been deposited in Gene Expression Omnibus (GEO) under accession # GSE13292.

Exploratory data analysis was performed on probe cell intensities and summarized probe set signal values. Box plots of signal distributions were generated with Expression Console (Affymetrix). Scatter plots and correlation measurements were performed using Statistica 6.0 (StatSoft, Tulsa, OK, USA). Venn diagrams were generated using VENNY, an interactive tool for comparing lists with Venn diagrams [21]

## Abbreviations

cDNA: complementary deoxyribonucleic acid; Ct: cycle threshold; CTP: cytidine triphosphate; DNA: deoxyribonucleic acid; GAPDH: glyceraldehydes-3-phosphate dehydrogenase; IVT: in vitro-transcription; MAS 5.0: Microarray Suite version 5.0; mRNA: messenger RNA; NF-κB: nuclear factor-kappa B; NK: natural killer; OHSU: Oregon Health and Science University; PCR: polymerase chain reaction; PNA: peptide nucleic acid; r: correlation coefficient; RIN: RNA integrity number; RMA: robust multi-array average; RNA: ribonucleic acid; RT-PCR: reverse transcriptase-polymerase chain reaction; UV: ultraviolet.

## Authors' contributions

KV performed the microarray assays and assisted with experimental design, data analysis and preparation of the manuscript; RS assisted with protocol development and microarray assays; AC assisted with protocol development and performed RNA isolations and globin RNA depletion; TJ performed data analysis; SP participated in the design of the study, data analysis and manuscript preparation; DC performed data analysis and participated in critical review of the manuscript; JS and JR participated in the design of the study and critical review of results and the manuscript; CAH conceived of the study, guided its design and coordination, performed data analysis and drafted the manuscript. All authors read and approved the final manuscript.
